# Isolated Tuberculosis of the Cervical Vertebrae

**DOI:** 10.7759/cureus.35383

**Published:** 2023-02-23

**Authors:** Amir A Mahmoud, Ali Abdelhay, Basant Eltaher, Mohamed S Mohamed

**Affiliations:** 1 Internal Medicine, Rochester Regional Health, Rochester, USA; 2 Hematology and Bone Marrow Transplant, Ain Shams University, Cairo, EGY

**Keywords:** cervical vertebrae, spinal mass, extrapulmonary tuberculosis (eptb), spinal mri, spine tuberculosis

## Abstract

Tuberculosis (TB) is a highly infectious disease that primarily affects the lungs, but extrapulmonary affection can occur with lymphatic or hematogenous spread. Skeletal affection commonly involves the spine, but cervical vertebral affection is rare. We report a 23-year-old female patient who presented to the hospital with diffuse limb weakness and neck pain as the only complaints. MRI of the cervical spine revealed a peripherally enhancing lesion arising from the posterior aspects of the cervical vertebrae with compressive myelopathy. She underwent surgical decompression and was noted to have caseous drainage during the procedure. She was started promptly on anti-tuberculous therapy after she had a positive interferon-gamma release assay. Late culture results confirmed isolated cervical TB of the vertebrae as the diagnosis. Prompt awareness and initiation of treatment for vertebral TB are necessary as clinical presentation can mimic other infectious and malignant etiologies.

## Introduction

Tuberculosis (TB) is a highly infectious disease that primarily affects the lungs, but extrapulmonary affection can occur with lymphatic or hematogenous spread. Skeletal affection commonly involves the spine, but cervical vertebral affection is rare. In this article, we present a rare case of isolated TB of the cervical vertebrae and subsequent caveats in clinical management.

## Case presentation

**Figure 1:**
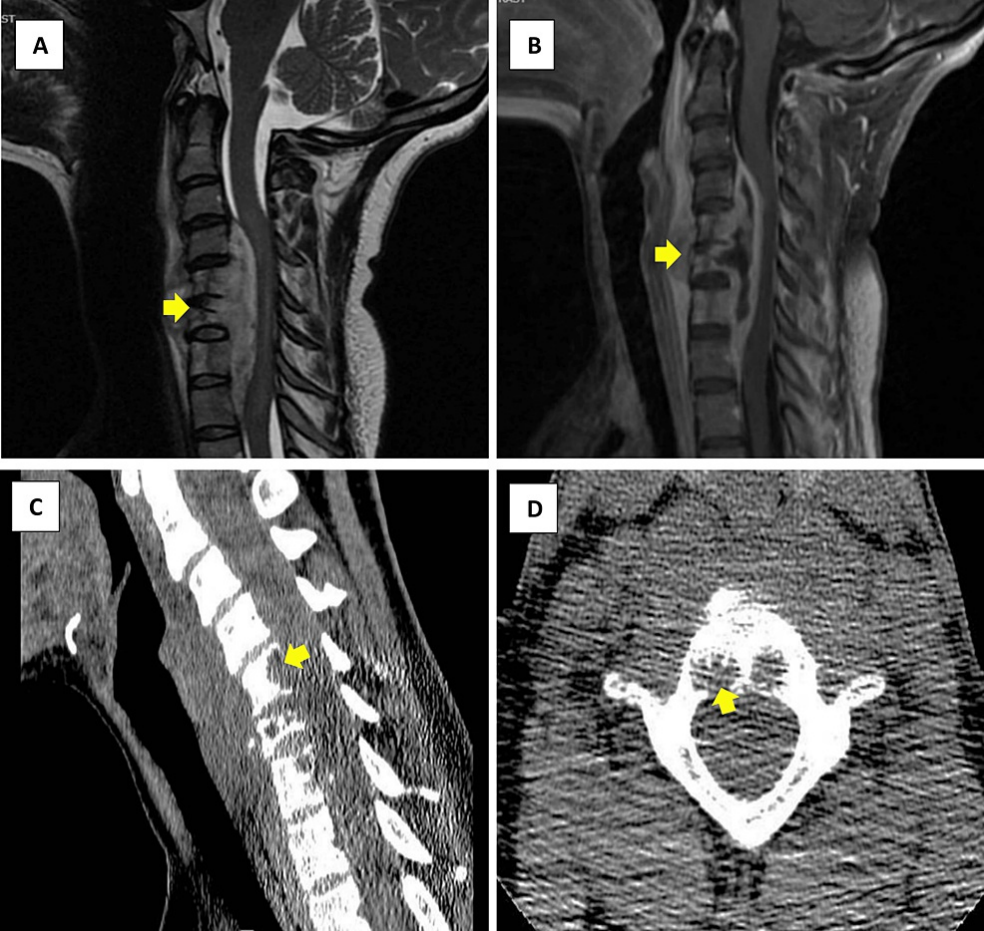
MRI and CT images of the cervical spine Images A and B show peripherally enhancing mass-like lesions (arrows) involving C5, C6, and C7 with significant spinal canal stenosis on MRI of the cervical spine. Images C and D show sagittal and axial cuts, respectively, of the mass-like lesions on the CT scan of the neck.

A 23-year-old female patient presented to the hospital with diffuse limb weakness. She noticed progressive weakness and paresthesia that started in her left arm three weeks prior and then progressed to involve her right arm and both lower limbs. The severity of her weakness has progressed to the point that she had difficulty in ambulation and had constant numbness and tingling in her hands and feet. She also had a history of dull aching neck pain for a one-year duration for which she underwent physical therapy without much relief. She denied any fever, weight loss, or change in appetite. She is originally from East Africa and immigrated to the United States five years ago. She denied previous exposure to TB or lung disease. An extensive review of systems was otherwise unremarkable. Vital signs were within normal ranges. Neurological examination was remarkable for marked symmetrical weakness, more pronounced in the upper extremities. Deep tendon reflexes were brisk, and sensory examination was normal. Cervical lymphadenopathy was also noted. Laboratory investigations showed mild leukopenia with monocytosis and iron deficiency anemia but were otherwise unremarkable. A chest x-ray (CXR) showed clear lung fields. MRI of the cervical spine (Figure 1, Panels A and B) revealed peripherally enhancing, centrally non-enhancing lesions arising from the posterior aspects of the C5 and C6 vertebral bodies with cranial and caudal extension from C3-C4 level to the T1 level as well as anterior extension into the prevertebral region and relative sparing of the intervertebral discs.

The lesion resulted in severe spinal canal stenosis which is most significant from the C4-C5 level through the C7 level with faint T2 hyperintense intramedullary signal within the spinal cord at this level, suggestive of compressive myelopathy. Corresponding osteolytic lesions were also seen on CT of the neck (Figure 1, Panels C and D) in C5-C6. She was treated with dexamethasone and underwent anterior cervical corpectomies and decompression with cervical spinal fusion from C2 to T3. Operative findings were notable for purulent effluence on the entrance to the spinal canal, and intra-operative pathology showed necrotizing granulomas with a lymphocytic background.

Interferon-gamma release assay was positive, and she was started empirically on anti-tuberculous therapy. Full pathology results showed scattered loose aggregates of epithelioid histiocytes consistent with granulomas, associated with extensive necrosis, occasional Langhans-type giant cells, and calcifications. Scattered small lymphocytes were seen in the background consisting mainly of CD3- and CD5-positive T cells and a minor component of CD20-positive B cells, compatible with a reactive lymphoid population. There was no evidence of lymphoid or epithelial malignancy on immunohistochemistry and flow cytometry. The histochemical stains for fungal and acid-fast organisms (AFB) were negative. After two weeks of incubation, surgical cultures were positive for TB, thus confirming extrapulmonary TB of the cervical vertebrae. The patient was treated with four-drug regimens of rifampin, isoniazid, pyrazinamide, and ethambutol plus vitamin B6, and was showing good neurological recovery before hospital discharge. At the six-month follow-up, she has near-normal strength in the upper limbs with the recovery of functional status and normal gait.

## Discussion

TB is a highly infectious disease that primarily affects the lungs. Infected patients develop either primary active disease or latent infection. In 2018, there were nearly 10 million newly diagnosed cases of TB globally [1]. Extrapulmonary TB is a common occurrence with a prevalence of 17.4% in the United States. The most common extrapulmonary sites affected are the bones and joints, with 50% of skeletal affection involving the spine [2,3]. Spinal TB, secondary to hematogenous spread, has been more prevalent in developing countries and seen in patients between the ages of 30 and 40. The most affected site is the thoracolumbar junction and less frequently the cervical spine (3%-5%) [3].

Cervical TB is of major concern due to its proximity to major vessels and increased liability for neurological complications. Direct spinal compression can lead to permanent neurological deficits and impaired quality of life if not identified and treated early. Patients often present with neck pain and restricted neck movement, as seen in our case, along with constitutional symptoms. Diagnosis of spinal TB involves laboratory workup, including erythrocyte sedimentation rate (ESR) and C-reactive protein (CRP), along with imaging and obtaining a tissue biopsy for histopathology and mycobacterial culture. While a positive interferon-gamma release assay can be sensitive and specific for TB, it might not differentiate active versus latent TB, especially since isolated extrapulmonary involvement presents a wider differential. Nonetheless, it can support the suspicion of TB and prompt the early start of treatment, as was done in our case. MRI is the preferred diagnostic imaging, which often shows a smooth-walled abscess with late involvement of the vertebral disc [4]. Cervical TB can be challenging to diagnose owing to the wide differential available. For instance, cervical Langerhans cell histiocytosis is rare but very similar in clinical and radiological presentation to cervical TB as it may also be complicated by vertebral destruction and abscess formation [5]. Lymphoma is another condition that may affect the cervical spine in which T2 sagittal MRI cervical spine exhibits vertebral destruction [6]. Though less common, other infectious pathologies mimicking vertebral TB in immunocompromised patients include brucellosis and epidural abscesses, e.g., *Staphylococcus aureus* infection [7,8].

Management of cervical TB typically involves the administration of quadruple therapy for two months followed by seven to nine months of double or triple therapy. Treatment should be started promptly on suspicion of TB as diagnostic mycobacterial culture growth can be delayed for weeks, as seen in our case [4]. Surgical intervention could be necessary to relieve severe neurological affection, in case of spine instability, or to provide debridement [9]. Intraoperatively, our patient had complete involvement and destruction of C6 from the inflammation, necessitating complete removal of the C6 vertebral body.

## Conclusions

TB of the cervical spine is a rare entity and can present in isolation of pulmonary affection, mimicking other infectious or malignant etiologies. Prompt treatment initiation is necessary and should not be withheld until culture data results.
